# Histology Assessment of Chitosan–Polyvinyl Alcohol Scaffolds Incorporated with CaO Nanoparticles

**DOI:** 10.3390/molecules30020276

**Published:** 2025-01-12

**Authors:** Carlos David Grande-Tovar, Jorge Ivan Castro Castro, Lemy Vanessa Barba-Rosado, Paula A. Zapata, Daniel Insuasty, Carlos-Humberto Valencia-Llano

**Affiliations:** 1 Grupo de Investigación de Fotoquímica y Fotobiología, Universidad del Atlántico, Carrera 30 Número 8-49, Puerto Colombia 081008, Colombia; lbarba@mail.uniatlantico.edu.co; 2 Tribology, Polymers, Powder Metallurgy and Solid Waste Transformations Research Group, Universidad del Valle, Calle 13 No. 100-00, Cali 760001, Colombia; jorge.castro@correounivalle.edu.co; 3 Grupo de Polímeros, Facultad de Química y Biología, Universidad de Santiago de Chile, Santiago 9170020, Chile; paula.zapata@usach.cl; 4 Departamento de Química y Biología, División de Ciencias Básicas, Universidad del Norte, Km 5 Vía Puerto Colombia, Barranquilla 081007, Colombia; insuastyd@uninorte.edu.co; 5 Grupo Biomateriales Dentales, Escuela de Odontología, Universidad del Valle, Calle 4B # 36-00, Cali 760001, Colombia; carlos.humberto.valencia@correounivalle.edu.co

**Keywords:** biocompatible, chitosan, calcium oxide nanoparticles, subdermal tissue regeneration

## Abstract

Scaffolds for regenerative therapy can be made from natural or synthetic polymers, each offering distinct benefits. Natural biopolymers like chitosan (CS) are biocompatible and biodegradable, supporting cell interactions, but lack mechanical strength. Synthetic polymers like polyvinyl alcohol (PVA) provide superior mechanical strength and cost efficiency but are not biodegradable or supportive of cell adhesion. Combining these polymers optimizes their advantages while adding metal oxide nanoparticles like calcium oxide (CaO NPs) enhances antimicrobial properties by damaging bacterial membranes. In this study, we obtained the formation of CaO NPs by calcinating eggshells, which were mixed in a polymeric network of CS and PVA to obtain four different membrane formulations for subdermal tissue regeneration. The spherical nanoparticles measured 13.43 ± 0.46 nm in size. Their incorporation into the membranes broadened the hydroxyl bands in the Fourier transform infrared (FTIR) analysis at 3331 cm⁻^1^. X-ray diffraction (XRD) analysis showed changes in the crystalline structure, with new diffraction peaks at 2θ values of 7.2° for formulations F2, F3, and F4, likely due to the increased amorphous nature and concentration of CaO NPs. Additionally, higher CaO NPs concentrations led to a reduction in thermal properties and crystallinity. Scanning electron microscopy (SEM) revealed a heterogeneous morphology with needle-like structures on the surface, resulting from the uniform dispersion of CaO NPs among the polymer chains and the solvent evaporation process. A histological examination of the implanted membranes after 60 days indicated their biocompatibility and biodegradability, facilitated by incorporating CaO NPs. During the degradation process, the material fragmented and was absorbed by inflammatory cells, which promoted the proliferation of collagen fibers and blood vessels. These findings highlight the potential of incorporating CaO NPs in soft tissue regeneration scaffolds.

## 1. Introduction

The increase in accident rates, trauma, burns, and malformations, among others, often requires emergency treatment in which tissue replacement or repair is needed. Unfortunately, the availability of compatible organs and the high rejection rates cause a shortage of available tissues, so the patient can not replace or repair the damaged organs in many cases [1,2,3]. Current treatments include autologous, allogeneic, xenotransplantation, and prostheses. However, these approaches face limitations in compatibility, coagulation problems, and long-term efficacy [4].

In this regard, generating 3D porous scaffolds provides the appropriate environment to promote the transport of proteins and agents necessary to trigger tissue regeneration. These scaffolds are generally developed through inorganic and organic compounds with cells or growth factors to provoke a synergic effect between the components, improving physicochemical properties such as thermal, mechanical, and cellular properties such as biocompatibility and biodegradability required in many tissues, causing an increase in cellular response (adhesion and proliferative) [5]. According to the above, the polymeric matrix, whether natural or syntectic, serves as a molecular template and inorganic nanoparticle agents promote tissue regeneration [6].

Chitosan (CS) is a natural, polycationic, linear polymer derived from the partial deacetylation of chitin, which is the main component of the crustacean exoskeleton. It is a linear heteropolysaccharide composed of β-1,4-linked D-glucosamine and N-acetyl-D-glucosamine, which attracts much attention for its biocompatibility, biodegradability, antibacterial and antifungal properties [7,8]. Several studies have allowed the coupling of chitosan with various biomaterials such as hyaluronic acid, calcium phosphate, alginate, or other biomolecules that enable the possibility of making different scaffolds applied in tissue engineering [9,10].

However, CS exhibits the common structural drawbacks of polysaccharides, such as limited stability in physiological media and low solubility in different solvents, which is related to the hydrogen bonds in its chains [11]. In addition, its low mechanical properties for load-bearing applications and poor water vapor barrier limit its direct applicability. According to the above, synthetic polymers can offer greater functionality, reproducibility, high purity, and cost-effectiveness than natural polymers [12,13]. However, their main drawbacks include low degradation rates and limited cell adhesion and interaction compared to their natural counterparts. Combining synthetic and natural polymers has effectively addressed these limitations by incorporating specific ligands, proteins, and other molecules that improve biocompatibility and cellular responses [14]. Polyvinyl alcohol (PVA) is a hydrophilic, able to form cross-linked biodegradable, resistant to deformation under load, non-toxic, and highly biocompatible synthetic polymer with significant potential for biomedical applications. Additionally, its chain flexibility greatly enhances its versatility in this field despite its instability in aqueous environments [15,16].

Incorporating chitosan and PVA has demonstrated remarkable synergistic potential, increased porosity, and improved mechanical and chemical properties [17]. These enhancements are attributed to the intermolecular interaction derived from their chemical structure and physical properties. PVA is characterized by the formation of extensive hydrogen bonds in its hydroxyl groups [18]. At the same time, CS contains amino and hydroxyl groups in its structure, which act as proton acceptors and donors [19]. This enables the formation of hydrogen bond interactions between the hydroxyl groups of PVA and the amino group of CS in a composite composed of both polymers [20]. These intermolecular interactions, combined with electrostatic attractions and Van der Waals forces between the polymers, confer enhanced physicochemical and mechanical properties, such as tensile strength, toughness, and thermal stability in aqueous systems [21].

Dashtdar et al. evaluated whether mesenchymal stem cells (MSCs) seeded in a hydrogel based on the PVA–CS mixture could achieve cartilage healing comparable to an alginate-transplant model [22]. Their study found that the PVA–CS–MSC construct produced similar outcomes in a rabbit cartilage defect model, supporting its potential for clinical applications in cartilage repair. Peng et al. confirmed that PVA–CS hydrogel promoted strong adhesion and proliferation of rabbit bone marrow MSCs with low cytotoxicity and good physicochemical and mechanical properties, resulting in superior cartilage repair in a rabbit model [23]. On the other hand, CaO nanoparticles (CaO NPs) were shown to have antimicrobial and antifungal activity, where their activity is related to the production of oxygen radicals and the alkalinization of the solutions in which they are found, causing damage to bacterial membranes and cell death [24,25]. In this context, the synthesis of CaO NPs is environmentally friendly, as they can be produced from components derived from organic waste, making it an economical and sustainable approach [18]. Similar systems of polymeric blends with nanoparticles have been studied. Lishchynskyi et al. prepared and characterized a polymeric brush adhered to a glass surface based on poly (di (ethylene glycol) methyl ether methacrylate) (POEGMA), not embedded with calcium carbonate nanoparticles (CaCO_3_). They found that, according to the temperature-sensitive properties, POEGMA coating with CaCO_3_ nanoparticles acts as a biologically active substrate, making them a potential new platform for tissue engineering [26].

However, there are few reports on calcium oxide nanoparticle blends in polymers for the generation of scaffolds and their implantation in animal models. These investigations are essential as a first step toward replacing conventional medical devices. In this context, we propose the formulation of a CS/PVA polymeric blend designed to produce a synergistic effect that enhances both cell adhesion and proliferation and optimizes its mechanical properties. When this blend is combined with CaO NPs, the resulting membranes exhibit more excellent antimicrobial and antifungal activity. Therefore, CS/PVA/CaO NP membranes represent an innovative solution for tissue regeneration and contribute to advancements in medical device development. To validate this proposal, four formulations of CS/PVA/CaO NP membranes were developed to evaluate their biodegradable and biocompatible properties in subdermal tissue after 60 days of implantation in Wistar rats.

## 2. Results and Discussion

### 2.1. Characterization of CaO NPs

#### Size and Morphology of the CaO NPs

**Figure 1:**
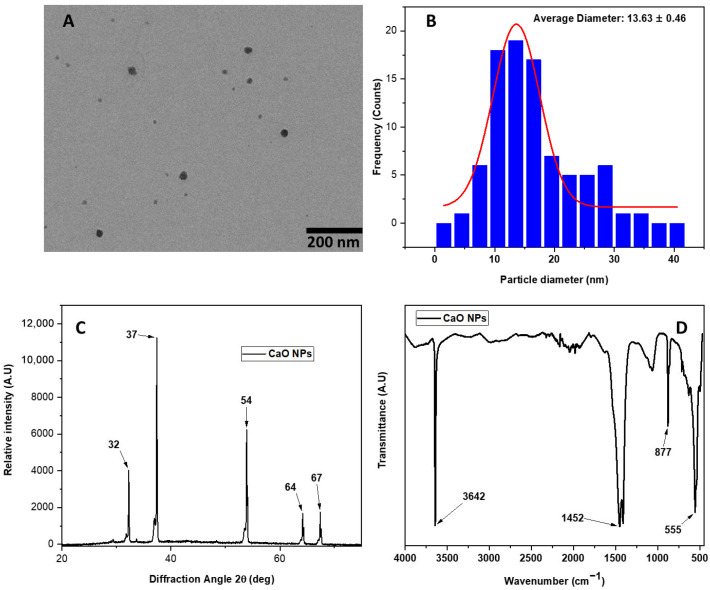
(**A**) TEM image, (**B**) histogram of CaO nanoparticles, (**C**) XRD pattern, and (**D**) FTIR spectra of CaO nanoparticles obtained from eggshells.

The process of characterization of the CaO NPs obtained by the milling process and thermal treatment with a yield of 97% is shown in Figure 1. The CaO NPs were analyzed by transmission electron microscopy (TEM), wherein Figure 1A,B show an average size of spherical nanoparticles of 13.43 ± 0.46 nm with a size distribution ranging from 1 to 40 nm. The crystalline structure is shown in the Figure 1C. The XRD diffraction pattern is observed in five signals at 32, 37, 54, 64, and 67° correspond to the crystallographic planes (001), (100), (101), (102), and (110) attributed to the portlandite phase (JCPDS 44–1481), respectively [27]. FTIR analyzed the presence of functional groups, and its spectra are shown in Figure 1D. The vibrational mode at 3642 cm^−1^ is related to the asymmetric vibration of the hydroxyl group (OH), associated with the water molecules from the air on the surface of the nanoparticles [28,29]. Meanwhile, the bands at 1487 cm^−1^ and 877 cm^−1^ correspond to the band identified as the asymmetric bond of the carbonate group. Finally, the high-intensity band around 500 cm^−1^ is associated with the vibration of the Ca–O bonds in the nanoparticles [30].

### 2.2. FTIR Analysis of CS/PVA/CaO NP Membranes

**Figure 2:**
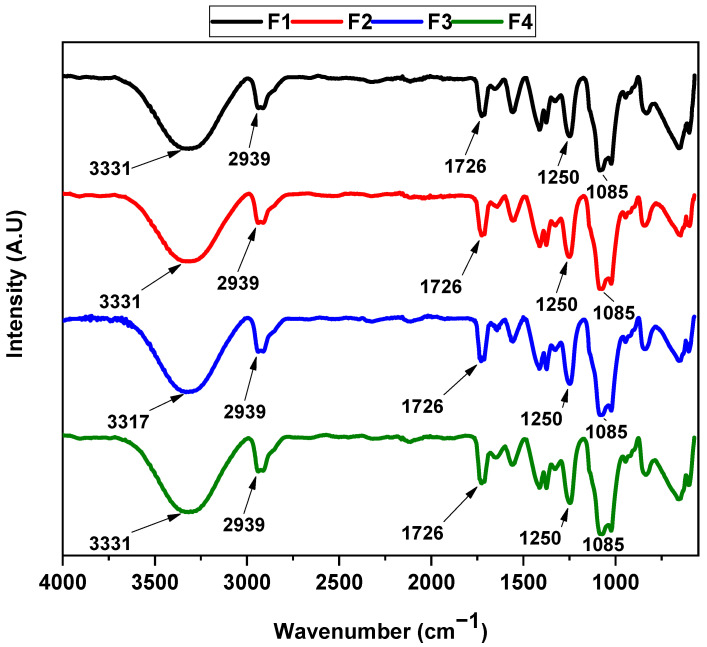
FTIR spectrum of PVA/CS/CaO NP membranes. F1, 30% CS/70% PVA; F2, 28% CS/70% PVA/2% CaO-NPs; F3, 26% CS/70% PVA/4% CaO-NPs; F4, 24% PCL/70% PLA/6% CaO-NPs.

The presence of the different functional groups for PVA/CS/CaO NP membranes is shown in Figure 2. Generally, for the materials F1, F2, F3, and F4, it was possible to observe one vibrational model symmetric at 3331 cm^−1^, which is attributed to the -OH groups present in both PVA and CS, consistent with observations in previous studies [31]. Furthermore, the vibrational modes symmetric and asymmetric for the -CH and -CH_2_ groups were observed at 2939 cm^−1^. In addition, the vibration mode at 1726 cm^−1^ was related to the presence of carbonyl group (C=O) type amido I and II of the CS. The vibrational mode at 1411 cm^−1^ corresponded to oscillations of OH and CH groups. Finally, it was possible to observe the vibrational mode attributed to the ester group (C-O-C) at 1250 cm^−1^, and at 1050 cm^−1^, the stretching vibrational mode of the C-O was observed.

F2, F3, and F4 have no significant differences in the formulations. Additionally, it was impossible to observe the presence of the vibrational mode for the CaO NPs at 560 cm^−1^. Nevertheless, when the amount of the CaO NPs increased, the broadening and shift of the –OH stretching vibration band from 3331 cm⁻^1^ to 3317 cm⁻^1^ likely resulted from hydrogen-bonding interactions between CaO NPs and CS/PVA. This interaction is crucial in ensuring the uniform distribution of CaO NPs within the polymer matrix, preventing agglomeration. Additionally, the shift of the bands related to the hydroxyl groups in the formulations, especially in F3, confirms the incorporation of CaO NPs within the CS–PVA polymeric matrix.

### 2.3. XRD of the CS/PVA/CaO NP Membranes

**Figure 3:**
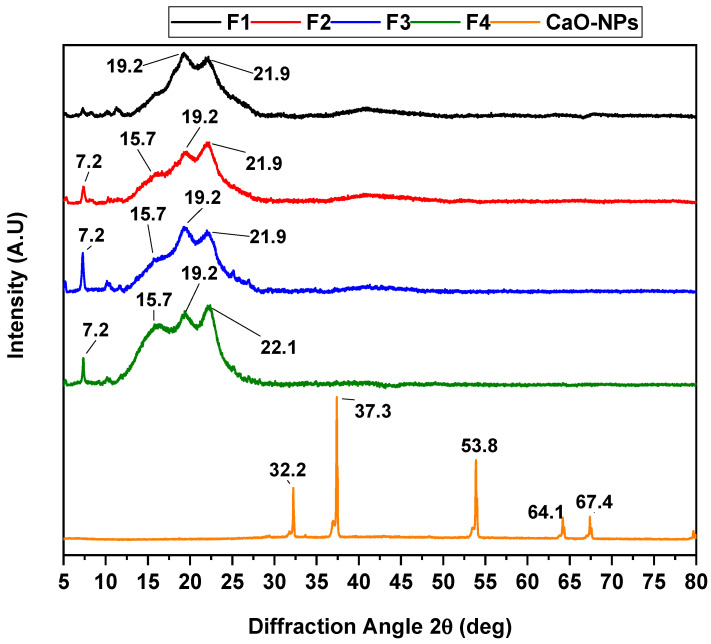
XRD diffractogram of CS/PVA/CaO NP membranes. F1, 30% CS/70% PVA; F2, 28% CS/70% PVA/2% CaO NPs; F3, 26% CS/70% PVA/4% CaO NPs; F4, 24% CS/70% PVA/6% CaO NPs.

The different peaks of diffractions for the PVA/CS/CaO NP membranes are shown in Figure 3. In the diffractogram, the predominant characteristic of each material is its amorphous nature, with small crystalline peaks appearing. These peaks at 19.2 and 21.9°, attributed to the 110 and 200 planes, represent the PVA monocyclic unity cell, which overlaps the peak characteristic of the CS at 19.8°. New diffraction peaks at 2θ values of 7.2° appeared in samples F2, F3, and F4, likely due to the membranes’ highly amorphous nature and increased CaO NPs. On the other hand, incorporating CaO NPs in formulations F2, F3, and F4 led to an increase in the intensity and an increase in the amplitude of the diffraction peaks at 2θ values of 15.7°. This suggests an interaction among the components CS, PVA, and CaO NPs without altering the crystallinity of the nanoparticles, as the characteristic peaks of the CaO NPs, shown in Figure 1C, exhibit a shift within the range of 30–70°.

### 2.4. Thermal Analysis of CS/PVA/CaO NP Membranes

**Figure 4:**
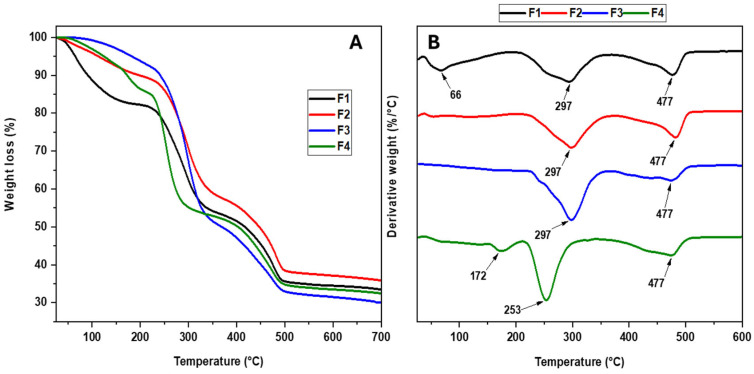
Thermogram (**A**) and its derivative curves (**B**) of the CS/PVA/CaO NP membranes. F1, 30% CS/70% PVA; F2, 28% CS/70% PVA/2% CaO NPs; F3, 26% CS/70% PVA/4% CaO NPs; F4, 24% CS/70% PVA/6% CaO NPs.

The thermograms and their derivatives for the CS/PVA/CaO NPs are shown in Figure 4. As the concentration of CaO NPs increases, the thermal stability of the membranes decreases, except for F2. Generally, the first stage of degradation between 60 and 100 °C corresponds to removing the physically absorbed water and partial dehydration of the PVA chains [32]. The second stage of degradation between 200 and 400 °C is attributed to the disintegration of the PVA side chain, i.e., the heating settling of the polymer composition of the PVA [33] and the related decomposition of the CS polysaccharide unit. Finally, the third stage corresponds to the degradation of the CS glycosidic bonds and the degradation of the PVA polyene residues [34]. Table 1 presents a summary of the different stages of degradation for each formulation.

Although there are no studies that relate the degradation by CaO NPs to polymers containing only hydroxyl groups, the only way to explain the degradation of the F3 and F4 formulations is the interaction between the carbonyl group of the CS with the CaO nanoparticles; moreover, it was shown that studies of nanocomposites between polylactic acid and CaO nanoparticles show a decrease in the thermal properties of the nanocomposites mainly due to the interaction between the C=O of the polylactic acid and CaO nanoparticles, causing depolymerization through the interaction of the ester-like carbonyl groups which causes the formation of oligomers with carboxylate and carboxylate end-groups. Then, the carboxylic ion binds to the asymmetric carbon atom in the penultimate unit of the polylactic acid, followed by the cleavage of the CO bond between the methine carbon and the oxygen of the ester [35,36]. In other words, the presence of a new stage of degradation for the formulation F4 (172 °C) may be related to the breaking of C-C and C=O bonds inside the polymeric matrix, especially in the CS, where the CaO NPs serve as crosslinking points, creating additional gaps between the polymers and reducing the structural order imposed by the intermolecular bonds. This observation is seen for the ZnO NPs with a polylactic acid-like polymeric matrix [37].

**Figure 5:**
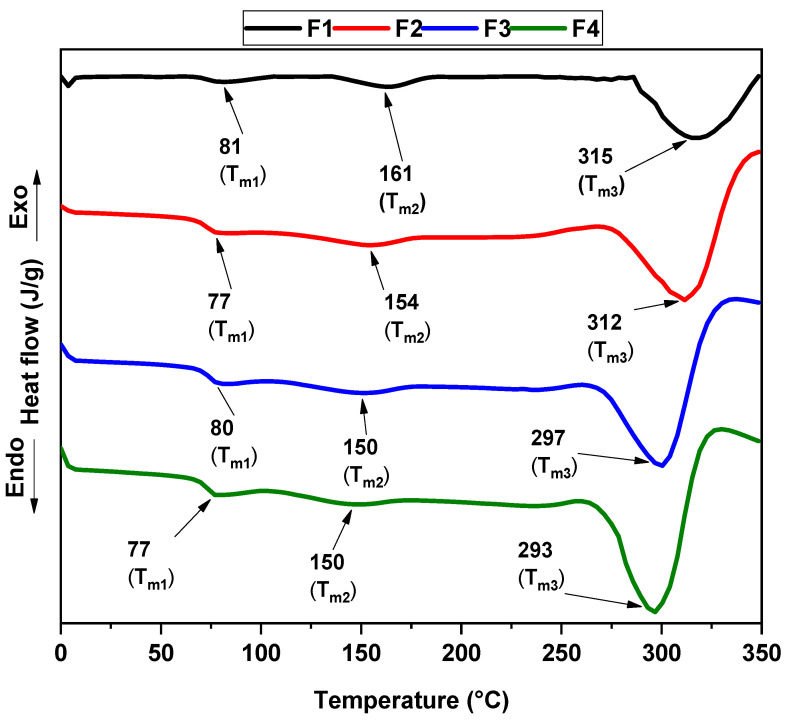
DSC thermograms of the CS/PVA/CaO NP membranes. F1, 30% CS/70% PVA; F2, 28% CS/70% PVA/2% CaO NPs; F3, 26% CS/70% PVA/4% CaO NPs; F4, 24% CS/70% PVA/6% CaO NPs.

Figure 5 presents the DSC thermograms of CS/PVA/CaO NPs. This thermogram determined the glass transition temperature Tg, melting temperatures Tm_1_ and Tm_2_ of PVA, and crystallinity percentage X_c_ for each formulation, as shown in Table 2, with results comparable to previous studies [38].

Additionally, the increase in CaO NPs is related to the decrease in the Tg of the membranes of CS/PVA/CaO NPs. The above is probably due to the aggregation of the CaO NPs, which weakens the polymeric network strength and intermolecular connection between PVA chains [33,39]. From the DSC curves, the crystallinity index is calculated to comprehend the changes in the polymer structure caused by the addition of CaO NPs. The following equation allows for the evaluation of the degree of relative crystallinity of the PVA [40]:

(1) $$X_{c}=\frac{\left(\Delta H_{m}\right)}{\Delta H_{m}^{{}^{\circ}}(1-x)}\times 100$$

where *X_c_* is the degree of crystallinity. $\Delta H_{m}$ is the enthalpy of melting that is determined through the DSC curves, and $\Delta H_{m}^{{}^{\circ}}$ is the enthalpy of 100% crystalline PVA melting, which is 138.6 J/g [41], and the wt. % of PVA is given by 1 - *x* term. The crystallinity index for the membranes is determined in Table 2. It is seen that the crystallinity of PVA/CS/CaO NPs increases with a small amount until 4% in wt, F3. However, the degree of crystallinity decreases when the quantity of CaO NPs is increased, F4. An increasing degree of crystallinity corresponds to reduced amorphization, whereas a decreasing trend indicates higher amorphization. This shift in crystallinity confirms the integration between PVA and CaO NPs, resulting in fewer contacts within the polymer network [42].

### 2.5. Scanning Electron Microscopy (SEM) of CS/PVA/CaO NP Membranes

**Figure 6:**
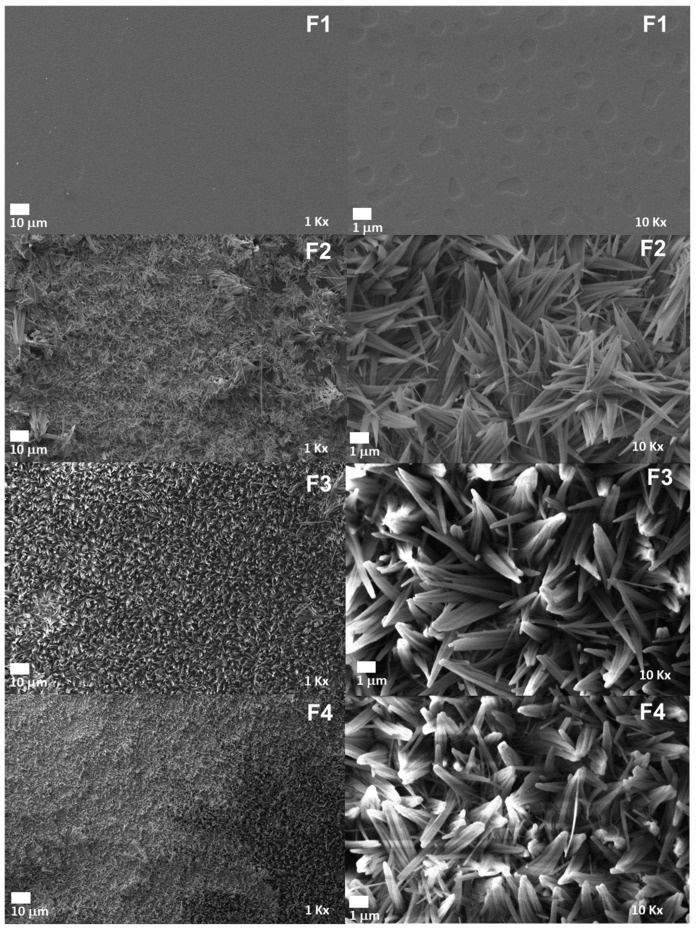
Morphology of PVA/CS/CaO NP membranes by SEM. F1, 30% CS/70% PVA at 1000× and 10,000×; F2, 28% CS/70% PVA/2% CaO NPs at 1000× and 10,000×; F3, 6% CS/70% PVA/4% CaO NPs at 1000× and 10,000×; F4, 24% PCL/70% PLA/6% CaO NPs at 1000× and 10,000×.

Figure 6 displays the electronic micrographs of the four membranes. The surfaces of these membranes are not smooth due to the interaction between the CaO nanoparticles and the CS/PVA polymeric matrix, where the CaO NPs are probably supported on the C=O groups of the chitosan, leading to reduced elasticity in the PVA chains owing to insufficient interfacial interactions between the nanoparticles and the polymeric matrix in the membranes. This could explain the emergence of new morphologies, which act as potential nucleation sites. These observations agree with investigations carried out with ZnO NPs in a polymeric matrix of polylactic acid [43].

On the other hand, only F1 exhibits a smooth surface, although some holes are visible at 10,000× magnification. This smoothness may be attributed to phase separation between the two polymers. The needle-like structures could result from the crystallization of polymer chains during the evaporation process involved in film formation. The morphology observed here is conducive to cell adhesion and proliferation during tissue regeneration, promoting degradation and reabsorption processes [44,45].

### 2.6. In Vivo Biocompatibility Tests of the CS/PVA/CaO NP Membranes

**Figure 7:**
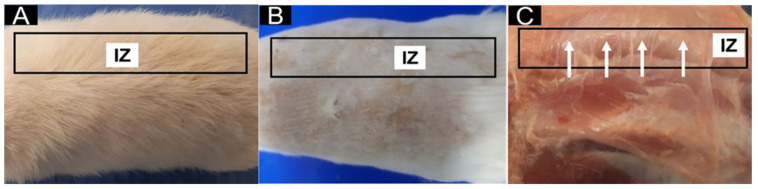
Macroscopic image of the dorsal surface implantation area in Wistar rats. (**A**): Biomodel with dorsal hair. (**B**): Dorsal surface with trichotomy. (**C**): Dorsal surface, internal aspect. Black rectangle: Intervened area. IZ: Implantation area. White arrows: Sites where specimens were implanted.

After confirming the euthanasia of the biomodels, a visual inspection was conducted on the areas where materials had been implanted. Hair recovery was observed on the dorsal surface, as illustrated in Figure 7A. Following a trichotomy performed on the biomodels, the healing of the pockets created during the procedure was evident, with no signs of infection (Figure 7B). Upon examining the internal skin area, it was noted that the treated regions had healed well, with the sites of the implanted samples appearing nearly invisible.

After processing the samples, histological analyses were conducted. It was observed that after 30 days, the tissues displayed signs of chronic inflammation, characterized by a foreign body reaction and the formation of a fibrous capsule in the samples where membranes created with the F1 formulation were implanted. Additionally, an inflammatory infiltrate was present in all formulations. Furthermore, the degree of resorption of the membranes varied depending on the formulation used.

**Figure 8:**
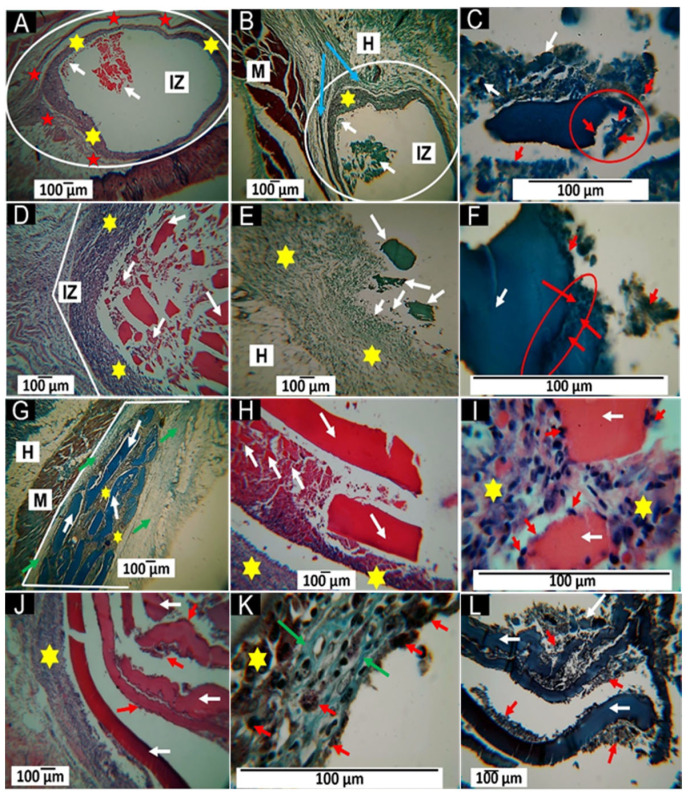
Samples were implanted subdermally for 30 days. **A**–**L** correspond to formulation F1; **D**–**F** correspond to formulation F2; **G**–**I** correspond to formulation F3 and **J**–**L** correspond to formulation F4. (**A**): 4× image, HE technique. (**B**): Image at 4×, GT technique. (**C**): Image at 40×, MT technique. (**D**): Image at 10×, HE technique. (**E**): 10× image, GT technique. (**F**): 100× image, MT technique. (**G**): 4× image, MT technique. (**H**): 4× image, HE technique. (**I**): 100× image, HE technique. (**J**): 4× image, HE technique. (**K**): 100× image, GT technique. (**L**): 4× image, Mt technique. White circle: magnetization zone. IZ: Implantation zone. Red star: Fibrous cap. Yellow star: Inflammatory infiltrate. White arrow: Implanted material. Blue arrow: type III collagen fibers. M: Muscle. H: Hypodermis. Red circle: area of interest where fragments of the material are being detached. Red arrow: inflammatory cells. Red oval: area of interest where the material is cracking. Green arrow: type I collagen.

Formulation F1 shows high resorption, as seen in Figure 8A,B. The implantation zone appears surrounded by fibrous tissue corresponding to a thin capsule (red stars in Figure 8A). Employing the Masson trichrome (MT) technique, the presence of type III collagen fibers is evident (blue arrows in Figure 8B). An inflammatory infiltrate surrounds the implantation zone (yellow stars). Figure 8C shows how different fragments of the material have separated and are attacked by phagocytic cells (red circle).

Figure 8D–F show the results for formulation F2, showing the fibrous capsule absence and the inflammatory infiltrate presence. Figure 8D shows many fragments of different sizes corresponding to the implanted material surrounded by the inflammatory infiltrate. Figure 8E also shows that the smaller particles are incorporated in the inflammatory infiltrate. Figure 8F shows a particle of the material in the process of fragmentation due to cellular activity, with the formation of a crack on its surface (red oval).

The results for the F3 formulation are very different from those shown for F1 and F2; the entire implantation zone was occupied by a connective tissue matrix with a significant presence of type I collagen, as shown in Figure 8G processed by the MT technique. In this image, fragments of the material that are much more important than those observed in the other formulations can also be seen; in the middle of these fragments is a brown tissue that seems to correspond to an inflammatory infiltrate.

Figure 8H corresponds to another biomodel; there are large fragments similar to those observed in Figure 8H, but an inflammatory infiltrate surrounds tiny fragments. Figure 8I, at a magnification of 100×, shows two small pieces, surrounded by the inflammatory infiltrate (yellow stars) and with phagocytic cells on their surfaces, indicated with red arrows.

Figure 8J–L correspond to the results of the F4 samples. The histological images are similar to the results for the F3 samples in that there is an absence of a fibrous capsule and an inflammatory infiltrate in the periphery of the implantation zone. However, the samples present less fragmentation, as seen in Figure 8J; in the peripheral zone where the inflammatory infiltrate is present, type I collagen fibers and numerous inflammatory cells can be identified using the MT technique (Figure 8K). Figure 8L corresponds to the inside of the implantation zone, where numerous fragments of the material in the fragmentation process by cellular action can be observed.

A comparison of the histological results of the four types of membranes shows differences that might be due to their chemical composition. F1 formulation is composed onñy of 30% CS/70% PVA and showed the highest resorption. The component materials of the other membranes are based on CS, PVA, and CaO NPs, all compatible and biodegradable materials. The samples composed only of CS and PVA showed higher resorption than the others. Chitosan is a biocompatible and biodegradable material, with enzymatic degradation being the primary mechanism by which removal takes place when implanted in living tissues [46].

In subdermal implantations of chitosan hydrogels, the material was highly degraded at 30 days with a deficient inflammatory response [47]. To improve the resorption rate of chitosan, PVA is added, which has a slower resorption but also improves the mechanical properties [48]. The finding of a fibrous capsule is prevalent when materials are implanted subdermally, and it is considered how the organism acts to limit the damage the material can cause. At the same time, it is resorbed, and the healing process is completed, which is the last phase of the foreign body reaction process [49]. It is well known that the properties of a material and its chemical composition influence biocompatibility and the type and duration of the inflammatory response [50]. The presence of such a thin capsule, as shown in Figure 8A, may be related to the resorption of the material because the capsule persists as long as there are remnants of the implanted material [51].

On the other hand, an inflammatory infiltrate is evident in all the implanted samples due to the chronic inflammation established to remove the implanted material, which will persist through the remnants [50]. A key observation is that as the percentage of chitosan (CS) decreases and the concentration of CaO nanoparticles (NPs) increases, the membranes exhibit less resorption. This trend is illustrated in the images presented in Figure 8. In Figure 8D, the material appears highly fragmented, while Figure 8G shows membranes undergoing a fragmentation process. By Figure 8J, the membranes display significant stability. CS (chitosan) has the highest resorption rate among the components, so the membranes exhibit excellent stability against degradation by reducing their presence. Adding CaO nanoparticles (NPs) enhances biocompatibility, as evidenced by the absence of a fibrous capsule observed in samples F2, F3, and F4. This improved biocompatibility may be attributed to the presence of CaO NPs, which are highly hygroscopic materials [52], despite the higher membrane degradation.

In a typical scarring process, the first few days are characterized by an acute inflammation phase, during which conditions are created to heal the injured tissue. However, if any factor disrupts this process, it may lead to a chronic inflammation phase [53]. When biomaterials are implanted, they can trigger a foreign body reaction. Fortunately, chronic inflammation associated with biocompatible materials is generally localized to the site of the material and typically lasts for a short duration [54]. This might explain why membranes made solely from CS/PVA exhibit a fragile capsule (Figure 8A,B). In experiments involving the implantation of membranes from the F2 formulation, the introduction of CaO nanoparticles resulted in the disappearance of the fragile capsule. Instead, a moderate inflammatory infiltrate was observed (Figure 8D,F), contributing to the membrane fragmentation and phagocytosis.

The presence of collagen type III and type I fibers is related to the maturation of the temporary matrix that is formed in the acute phase of the healing process; in the absence of irritating factors, the temporary matrix is composed mainly of fibrin [51], is replaced by collagen type III fibers, which are progressively replaced mainly through collagen type I fibers [48]. Figure 8G,K show the results of the implantations of membranes F3 and F4 with a higher percentage of CaO NPs, observing a change in the inflammatory infiltrate from moderate to mild; a collagen type I fiber matrix supports the inflammatory cells.

## 3. Materials and Methods

### 3.1. Materials

All precursors used in this research were of reagent grade. PVA with a hydrolysis grade of 87–89% and an Mw 93,000 g/mol was purchased from Sigma-Aldrich (Palo Alto, CA, USA). Glacial acetic acid from Merck (Burlington, MA, USA) was used to prepare CS solutions. A capillary viscometer was used to determine the molecular weight of chitosan (CS). At the same time, gel permeation chromatography (GPC) was applied to measure the viscosity average molecular weight (Mv) using viscous CS solutions. Drop times of 25 mL solutions, prepared from a standard solution, were recorded using an Ubbelohde viscometer.

The intrinsic viscosity ([*η*]), representing the intercept of the specific viscosity curve, was calculated using the Houwink–Sakurada equation [55], with constants *K* (0.074 mL/g) and *a* (0.76), the *Mv* of CS was found to be 144,000 Da, in a solution of 0.3 M acetic acid and 0.2 M sodium acetate at 25 °C.

(2) $$\left[\eta \right]=K{\left(Mv\right)}^{a}$$

The grade of CS deacetylation was 89–90%, determined through 400 MHz ^1^H-NMR spectroscopy using a BRUKER AVANCE II (Bruker, Berlin, Germany) with a temperature control of 300 °K in D_2_O. A Thermo Electron Flash EA 1112 elemental analyzer (Thermo Fischer, Waltham, MA, USA) was utilized to determine the composition of the elements. Finally, a domestic eggshell was used to synthesize calcium oxide nanoparticles.

#### 3.1.1. Synthesis of CaO NPs

**Figure 9:**
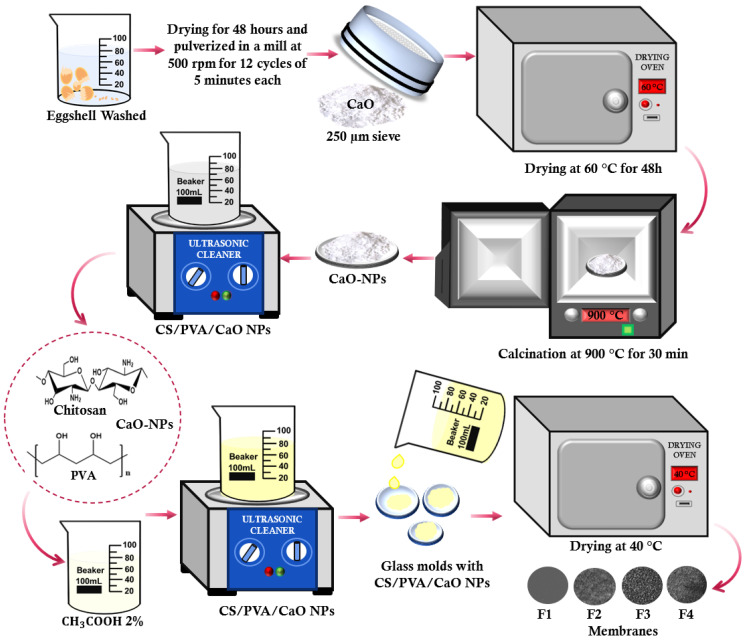
Methodology for the synthesis of CaO NPs and the preparation of CS/PVA/CaO NP membranes.

Previous methodologies carried out the synthesis of the CaO NPs [45,56]. Initially, eggshells were collected, washed with abundant distilled water, and dried for 48 h. Subsequently, they were processed in a ball mill at 500 rpm for 12 cycles of 5 min. Then, they were passed through a sieve with a pore size of 250 microns and dried for 48 h at 60 °C. Finally, the nanoparticles were obtained by calcination at 900 °C for 30 min (Figure 9).

#### 3.1.2. Characterization of the CaO NPs

The morphology of the CaO NPs was analyzed by transmission electron microscopy (TEM) using an instrument JEOL ARM 200 F (Tokyo, Japan). A drop of the CaO NPs previously sonicated was deposited on a carbon-coated copper grid and dried at room temperature was added for study. An average of 100 nanoparticles was calculated using ImageJ software (version 1.54g; Java 1.8.0_345, 64-bit) for particle size calculation. Crystallographic diffraction planes related to CaO NPs were carried out on a PANalytical X0Pert PRO diffractometer (Malvern Panalytical, Jarman Way, Royston, UK) using Cu Kα1 (1.540598 Å) and Kα2 (1.544426 Å) radiation, with an accelerating voltage of 45 kV in a 2θ range between 5 and 80°. Finally, the functional groups corresponding to the vibrational modes of the CaO NPs were analyzed by infrared spectroscopy using Shimadzu-type equipment (Kyoto, Japan) in the attenuated total diffuse reflectance (ATR) mode.

### 3.2. Preparation of the CS/PVA/CaO NP Membranes

To prepare the different membranes, we considered a specific amount of solid totals present in each formulation of 4% (wt.%). Then, a dispersion of the CaO NPs (100 mg/10 mL) in H_2_O milli q was prepared in an ultrasonic bath (Branson, Madrid, Spain) for two hours. Subsequently, each component was a mixture in a solution of 2% CH_3_COOH tanking, as shown in Table 3. Finally, the mixture was transferred to an ultrasonic bath to eliminate the bubbles in the solution. Then, the varieties were transferred onto glass molds in a preheated oven at 40 °C ± 0.2 to synthesize the PVA/CS/CaO NPs.

### 3.3. Characterization of the CS/PVA/CaO NP Membranes

#### 3.3.1. Fourier Transform Infrared Spectroscopy and XRD Patterns

The different membranes’ diffractograms and FTIR spectra were obtained under the same conditions to characterize the CaO NPs.

#### 3.3.2. TGA and DSC Analysis

Thermal properties of the PVA/CS/CaO NP membranes were assessed using a NETZSCH TG 209 F1 Libra instrument from Mettler Toledo, (Schwerzenbach, Switzerland), with samples heated from 25 to 900 °C in an Al₂O₃ crucible at 10 °C/min under nitrogen. Differential scanning calorimetry (DSC) on a DSC1/500 instrument (Mettler Toledo, Schwerzenbach, Switzerland) analyzed thermodynamic parameters in a nitrogen atmosphere. A 9 mg cross-section of the membranes was heated from 25 to 250 °C at 10 °C/min, measuring the glass transition temperature (Tg) at the transition midpoint, melting temperature (Tm) at the endothermic peak, and crystallization temperature (Tcc) at the exothermic peak. Measurements were taken from the second heating cycle to eliminate the polymer thermal memory.

#### 3.3.3. Microstructure Studies

The morphology of PLA/n-CaO electrospun fibers was examined using a PHILIPS XL30 Scanning Electron Microscope (SEM) with 5000× resolution. These samples were analyzed using the secondary mode of electrons at 20 kV. A gold coating was added before the imaging to improve the material’s conductivity.

#### 3.3.4. In Vivo Biocompatibility Studies of the CS/PVA/CaO NPs

##### Surgical Preparation of Biomodels

This study evaluated the in vivo biocompatibility of four material formulations via subcutaneous implantation in biomodels, allowing multiple samples to be implanted at once and observed over time. The experiment followed UNE: 10993-6 (Biological evaluation of medical devices—Part 6: Tests for local effects after implantation. ISO 10993-6: 1994) guidelines for local effects after implantation. Five Wistar rats of five-month-old from the Universidad del Valle LABBIO lab were selected. After sedation, 1 cm × 10 cm pockets were made on the right side of each model dorsal midline, where blocks were implanted for preliminary biocompatibility testing [57].

After a 60-day implantation period, the biomodels were euthanized, and implant sites were prepared for analysis. Samples were stored in formalin, washed, dehydrated, diaphanized with xylol, and embedded in paraffin using a Leica Auto-technicon Tissue Processor™. The sample sections were then prepared for histological examination using the Thermo Scientific™ Histoplast Paraffin™ kit.

##### Histological Analysis

Paraffin-embedded tissue samples were sectioned to a 5 µm thickness with a Leica microtome and analyzed after 48 h on slides using hematoxylin-eosin (HE) and Masson trichrome (MT) staining. Histological images were captured with a Leica DFC 295 camera and DM750 microscope and analyzed with Leica Microsystems software (Mannheim, Germany) [57]. Ethical oversight was provided by the Biomedical Experimentation Animal Ethics Committee (CEAS) at Universidad del Valle, adhering to ARRIVE guidelines. There were no intraoperative or postoperative complications or animal deaths. Inclusion criteria focused on the animal’s sex, age, and weight, while welfare conditions served as discontinuation criteria. No control materials were used to minimize animal discomfort; instead, the aim was to compare responses to the four formulations.

## 4. Conclusions

In this study, we synthesized four CS/PVA/CaO NP membranes that demonstrated enhanced thermal stability compared to their components. The presence of CaO NPs was confirmed using a range of characterization techniques, including FTIR, XRD, TGA, and DSC. FTIR analysis revealed an increase in the broadening and a shift of the hydroxyl group wavenumber around 3300 cm^−1^, suggesting that CaO NPs primarily interact with this functional group. Additionally, as CaO NP concentration increased, a reduction in both thermal properties and crystallinity was observed until a certain degree of incorporation of the CaO NPs. SEM analysis further indicated a heterogeneous morphology with needles on the surface, attributed to the uniform dispersion of CaO NPs among polymer chains and the evaporation process of the solvent.

Moreover, the CS/PVA/CaO NP membranes exhibited preliminary biocompatibility, supporting tissue healing after degradation and resorption without triggering an aggressive immune response. During the degradation process, the material fragmented and was engulfed by inflammatory cells, promoting collagen fiber, blood vessels, and cell proliferation and further facilitating material resorption and biocompatibility. These findings highlight the potential of CaO NP-based scaffolds in promoting soft tissue regeneration.

## Figures and Tables

**Table 1:** Stages of degradation for the different formulations.

	F1	F2	F3	F4
60–100 °C	66	-	-	-
100–200 °C	-	-	-	172
200–400 °C	297	297	297	253
400–900 °C	477	477	477	477

**Table 2:** Thermal properties of the CS/PVA/CaO NP membranes. F1, 30% CS/70% PVA; F2, 28% CS/70% PVA/2% CaO NPs; F3, 26% CS/70% PVA/4% CaO NPs; F4, 24% CS/70% PVA/6% CaO NPs.

	*T_g_* (°C)	*T_m_*_1_ (°C)	*T_m_*_2_ (°C)	*T_m_*_3_ (°C)	$\Delta H_{m}$ (J/g)	*X_c_* PVA (%)
F1	41	81	161	315	80.4	17.4
F2	39	77	154	312	85.7	18.5
F3	37	80	150	297	199.7	43.2
F4	35	77	150	293	102.8	22.3

**Table 3:** Percentage composition (wt.%) for each PVA/CS/CaO NP membrane component.

Components	F1	F2	F3	F4
CS (%)	30	28	26	24
PVA (%)	70	70	70	70
CaO NPs (%)	0	2	4	6

## Data Availability

The original contributions presented in this study are included in the article. Further inquiries can be directed to the corresponding author.
